# Impact of Matrix Metalloproteinase-11 Gene Polymorphisms on Biochemical Recurrence and Clinicopathological Characteristics of Prostate Cancer

**DOI:** 10.3390/ijerph17228603

**Published:** 2020-11-19

**Authors:** Chun-Yu Hsieh, Ying-Erh Chou, Chia-Yen Lin, Shian-Shiang Wang, Ming-Hsien Chien, Chih-Hsin Tang, Jian-Cheng Lin, Yu-Ching Wen, Shun-Fa Yang

**Affiliations:** 1 Institute of Medicine, Chung Shan Medical University, Taichung 402, Taiwan; winnieling0608@gmail.com (C.-Y.H.); intointo814@gmail.com (Y.-E.C.); lcyhank.tw@gmail.com (C.-Y.L.); jianchenglin82318@gmail.com (J.-C.L.); 2 School of Medicine, Chung Shan Medical University, Taichung 402, Taiwan; sswdoc@vghtc.gov.tw; 3 Department of Medical Research, Chung Shan Medical University Hospital, Taichung 402, Taiwan; 4 Department of Surgery, Division of Urology, Taichung Veterans General Hospital, Taichung 407, Taiwan; 5 Graduate Institute of Clinical Medicine, College of Medicine, Taipei Medical University, Taipei 110, Taiwan; mhchien1976@gmail.com; 6 Department of Medical Education and Research, Wan Fang Hospital, Taipei Medical University, Taipei 116, Taiwan; 7 Department of Pharmacology, School of Medicine, China Medical University, Taichung 404, Taiwan; chtang@mail.cmu.edu.tw; 8 Chinese Medicine Research Center, China Medical University, Taichung 404, Taiwan; 9 Department of Biotechnology, College of Health Science, Asia University, Taichung 413, Taiwan; 10 Department of Urology, Wan Fang Hospital, Taipei Medical University, Taipei 110, Taiwan; s811007@yahoo.com.tw; 11 Department of Urology, School of Medicine, College of Medicine, Taipei Medical University, Taipei 110, Taiwan

**Keywords:** prostate cancer, MMP-11, polymorphism

## Abstract

Prostate cancer is among the most common malignant tumors worldwide. Matrix metalloproteinase (MMP)-11 is involved in extracellular matrix degradation and remodeling and plays an essential role in cancer development and metastasis. This study investigated the association of *MMP-11* polymorphisms with the clinicopathological characteristics and biochemical recurrence of prostate cancer. Five single-nucleotide polymorphisms (SNPs) of the *MMP-11* were analyzed in 578 patients with prostate cancer through real-time polymerase chain reaction analysis. A prostate-specific antigen level of >10 ng/mL, Gleason grade groups 4 + 5, advanced tumor stage, lymph node metastasis, invasion, and high-risk D’Amico classification were significantly associated with biochemical recurrence in the patients (*p* < 0.001). *MMP-11* rs131451 “TC + CC” polymorphic variants were associated with advanced clinical stage (T stage; *p* = 0.007) and high-risk D’Amico classification (*p* = 0.015) in patients with biochemical recurrence. These findings demonstrate that *MMP-11* polymorphisms were not associated with prostate cancer susceptibility; however, the rs131451 polymorphic variant was associated with late-stage tumors and high-risk D’Amico classification in prostate cancer patients with biochemical recurrence. Thus, the *MMP-11* SNP rs131451 may contribute to the tumor development in prostate cancer patients with biochemical recurrence.

## 1. Introduction

Prostate cancer is the second most frequently diagnosed cancer worldwide and is among the most common causes of cancer mortality among men [1,2]. In recent years, the incidence of prostate cancer has increased globally, even in Asian countries, where the incidence was reported to be low in the past [3]. Risk factors such as ethnicity, family history, diet, smoking, and somatic genomic alterations have been suggested to be associated with prostate cancer carcinogenesis [4,5]. The risk of prostate cancer increases with age in men, and most patients are diagnosed after the age of 65 years [6]. Currently, serum prostate-specific antigen (PSA) levels are used to diagnose, monitor, and evaluate prostate cancer. Patients with PSA levels above 10 ng/mL have a nearly 50% chance of developing prostate cancer. A higher PSA level indicates a greater risk of prostate cancer [7]. Moreover, PSA is a pivotal tool for determining the recurrence of prostate cancer. Specifically, the definition of biochemical recurrence (BCR) is associated with elevated serum PSA levels in patients with prostate cancer after treatment [8,9].

Matrix metalloproteinases (MMPs), also known as matrixins, are a family of calcium-dependent zinc-containing endopeptidases that can degrade extracellular matrix (ECM) proteins and aid in ECM remodeling; hence, they play a major role in the development and metastasis of cancer [10]. MMP-11, also named stromelysin-3 (SL-3), was first identified in stromal cells surrounding invasive breast carcinomas [11]. MMP-11 expression has been demonstrated to be upregulated in the serum and solid tumor tissues of patients with different types of cancer, such as non–small cell lung cancer [12], esophageal carcinoma [13], pancreatic carcinoma [14], ovarian carcinoma [15], colon cancer [16], and oral cancer [17]. However, MMP-11 expression is almost absent in normal tissues. Moreover, MMP-11 overexpression in patients with prostate adenocarcinoma was suggested to be associated with poor prognosis and survival [18].

A single-nucleotide polymorphism (SNP) is a common DNA sequence defined as a single-nucleotide variation (frequency, >1%) in the genome (or other shared sequences) [19]. Genetic polymorphisms in *MMP-11* have been reported in several types of cancer, including oral squamous cell carcinoma (OSCC) [20], breast cancer [21], hepatocellular carcinoma (HCC) [22], uterine cervical cancer [23], and urothelial cell carcinoma [24]. Our previous study revealed that *MMP-11* SNP rs738791 was associated with a greater risk of uterine cervical invasive cancer and HCC [22,23]. The HCC patients with at least one polymorphic C allele (C/T + C/C genotype) of *MMP-11* SNP rs738792 were prone to develop moderate to severe liver failure [22], and patients of OSCC with at least one polymorphic C allele of *MMP-11* rs738792 were found to be associated with an increased incidence of lymph node metastasis [20], compared with the homozygous T/T genotype. The *MMP-11* SNP rs28382575 was found that carriers with at least one polymorphic C allele (C/T + C/C genotype) were associated with a higher risk of developing large tumors, lymph node metastasis, or stage III/IV disease in HCC [22]. However, the impact of *MMP-11* polymorphisms on the risk and prognosis of prostate cancer remains poorly investigated. In this study, we analyzed five *MMP-11* gene polymorphisms (rs131451, rs738791, rs2267029, rs738792, and rs28382575) to elucidate their relationships with the clinicopathological characteristics and biochemical recurrence of prostate cancer.

## 2. Materials and Methods

### 2.1. Study Subjects

We enrolled 578 patients with adenocarcinoma of the prostate who underwent robotic-assisted laparoscopic radical prostatectomy at Taichung Veterans General Hospital in Taiwan from 2012 to 2017. Information about the initial PSA level at diagnosis, Gleason grade group [25], clinical and pathological tumor–node–metastasis (TNM) staging, Gleason score at initial biopsy, D’Amico classification [26], and other permanent pathological features were obtained from their medical records. The patients were staged according to the TNM staging system of the Eighth Edition of the American Joint Committee on Cancer (AJCC) staging manual [27]. This study was approved by the Institutional Review Board of Taichung Veterans General Hospital (IRB No. CE19062A; 04/March/2019), and informed written consent was obtained from each patient.

### 2.2. Specimen Collection and Genomic DNA Extraction

Peripheral blood specimens were collected from the patients before surgery. The specimens were placed in tubes containing ethylenediaminetetraacetic acid (EDTA), centrifuged, and then stored at −80°C. Genomic DNA was extracted from the buffy coats of the whole-blood specimens by using QIAamp DNA blood mini kits (Qiagen, Valencia, CA, USA) according to the manufacturer’s instructions. The final eluted DNA was dissolved in TE buffer (10 mM Tris and 1 mM EDTA; pH 7.8) and stored at −20°C before real-time polymerase chain reaction (PCR) analysis.

### 2.3. Selection of Matrix Metalloproteinase-11 Polymorphisms

**Figure 1:**
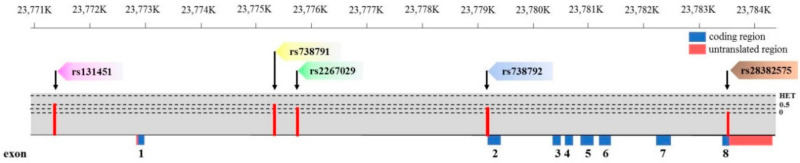
Exon and intron position of *MMP-11* gene in human and *MMP-11* gene polymorphisms assessed in the study.

Five *MMP-11* SNPs (rs131451, rs738791, rs2267029, rs738792, and rs28382575) with minor allele frequencies >5% were selected from the international HapMap project data for this study (Figure 1) [28]. The *MMP-11* intron variant rs738791 and nonsynonymous SNP rs738792 (exon 2, Ala38Val) were selected because these gene polymorphisms were suggested to be associated with a greater risk of uterine cervical invasive cancer and HCC [22,23]. The *MMP-11* SNP synonymous rs28382575 (exon 8, Pro475Pro) was selected because it was found that carriers with at least one polymorphic C allele (C/T + C/C genotype) were associated with a higher risk to develop large tumors, lymph node metastasis, or stage III/IV disease in HCC [22]. The *MMP-11* SNP rs131451 was selected because this gene polymorphism was thought to potentially provide tumor markers in urothelial cell carcinoma (UCC) treatment or predictors for UCC susceptibility and prognosis [24]. The intron variant rs2267029 was selected in this study as in previous cancer research [20].

### 2.4. MMP-11 SNP Genotyping Determination

Assessments of allelic discriminations for the MMP-11 rs131451 (assay ID: C___2213679_30), rs738791 (assay ID: C___2448099_30), rs2267029 (assay ID: C__15871447_20), rs738792 (assay ID: C___2213764_20), and rs28382575 (assay ID: C__61238655_10) SNPs were performed using the ABI StepOnePlus™ Real-Time PCR System. The ABI TaqMan^®^ SNP Genotyping Assay (Applied Biosystems; Foster City, CA, USA) was used for genotyping, according to the manufacturer’s protocols. The final data were collected and further analyzed using ABI StepOnePlus™ Software v2.3.

### 2.5. Statistical Analyses

The chi-square test and Student’s t-test were used to determine the differences in the distributions of the demographic characteristics of prostate cancer patients with or without biochemical recurrence. Odds ratios (ORs) along with their 95% confidence intervals (CIs) were estimated using logistic regression models to estimate the association between genotypic frequencies, biochemical recurrence, and different clinicopathological characteristics in patients with prostate cancer. Moreover, we estimated adjusted ORs along with their 95% CIs by using multiple logistic regression models after controlling for age at diagnosis, PSA levels at diagnosis, pathologic Gleason grade group, clinical T stage, pathologic T stage, pathologic N stage, seminal vesicle invasion, perineural invasion, lymphovascular invasion, D’Amico classification, and biochemical recurrence. *p*-values of less than 0.05 were considered statistically significant. All data were analyzed using SAS statistical software (version 9.1; SAS Institute, Cary, NC, USA) for Windows.

## 3. Results

The demographic characteristics of patients with prostate cancer are presented in Table 1. Of the 578 patients with prostate cancer, 175 were confirmed to present with biochemical recurrence. In addition to age at diagnosis, significant differences (*p* < 0.001) in PSA (at diagnosis), pathologic Gleason grade group, clinical T stage, pathologic T stage, pathologic N stage, seminal vesicle invasion, perineural invasion, lymphovascular invasion, and D’Amico classification were observed between the two groups (with or without biochemical recurrence) of patients.

The distribution frequencies of *MMP-11* genotypes in patients with prostate cancer are presented in Table 2. The genotypic distribution of *MMP-11* SNPs rs131451, rs738791, rs2267029, rs738792, and rs28382575 all conformed to this equilibrium in the prostate cancer patients (*p* = 0.191, χ^2^ value: 1.712; *p* = 0.504, χ^2^ value: 0.446; *p* = 0.126, χ^2^ value: 2.331; *p* = 0.109, χ^2^ value: 2.566 and *p* = 0.427, χ^2^ value: 0.632, respectively). The highest distribution frequencies of the *MMP-11* rs131451, rs738791, rs2267029, and rs28382575 polymorphisms were the heterozygous TC, homozygous CC, homozygous GG, and homozygous TT genotypes, respectively. The frequencies of the TT and TC genotypes were found to be the highest in the *MMP-11* rs738792 polymorphism. After adjustment for potential confounders, no significant differences in *MMP-11* rs131451, rs738791, rs2267029, rs738792, and rs28382575 SNPs were observed between prostate cancer patients with biochemical recurrence and those without biochemical recurrence.

To clarify the role of *MMP-11* gene polymorphisms in the clinicopathological characteristics of prostate cancer such as clinical staging, pathologic staging, pathologic Gleason grade group, invasion and D’Amico risk classification, the distribution frequencies of the clinicopathological characteristics and *MMP-11* genotypic frequencies in 578 patients with prostate cancer were estimated. As shown in Table 3, Table 4, Table 5, Table 6 and Table 7, we observed no significant associations between the *MMP-11* rs131451, rs738791, rs2267029, rs738792, and rs28382575 gene polymorphisms and the clinicopathological characteristics of the patients with prostate cancer.

We further analyzed the distribution frequencies of the clinicopathological characteristics and *MMP-11* genotypic frequencies in prostate cancer patients with biochemical recurrence. An analysis of the association between the *MMP-11* rs131451 polymorphism and patients with biochemical recurrence revealed significant differences in the clinical T stage and D’Amico classification (*p* = 0.007 and 0.015, respectively; Table 8). However, the *MMP-11* rs738791, rs2267029, rs738792, and rs28382575 polymorphisms were not significantly associated with the clinicopathological characteristics of patients with biochemical recurrence.

**Figure 2:**
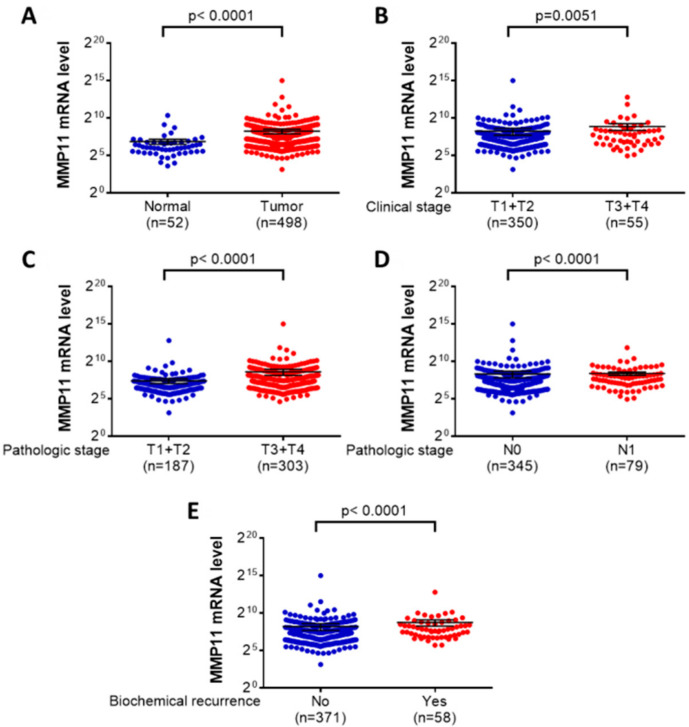
*MMP-11* mRNA level of patients with prostate cancer from the TCGA database. (**A**) *MMP-11* expression in 498 tumor tissues and the noncancerous tissues. (**B**) *MMP-11* mRNA levels were compared according to the clinical T stage status. (**C**) *MMP-11* mRNA levels were compared according to the pathological T stage. (**D**) *MMP-11* mRNA levels were compared according to the lymph node status. (**E**) *MMP-11* mRNA levels were compared according to the biochemical recurrence statuses.

We further used data from The Cancer Genome Atlas (TCGA) data set to analyze and clarify the findings of our study. The results of the TCGA data showed that there were statistical significant differences between the MMP-11 mRNA level and the patients with prostate cancer and normal controls (*p* < 0.0001), clinical T stage (*p* = 0.0051), pathological T stage (*p* < 0.0001), pathological N stage (*p* < 0.0001) and biochemical recurrence (*p* < 0.0001) (Figure 2).

## 4. Discussion

In this study, we examined the associations of *MMP-11* polymorphisms with the clinicopathological characteristics and biochemical recurrence of prostate cancer. A previous study suggested aging to be a major risk factor for prostate cancer, with more than 60% of patients diagnosed as having prostate cancer being aged older than 65 years [6]. In the current study, we observed no statistically significant difference in age at diagnosis between the prostate cancer patients with or without biochemical recurrence (*p* = 0.605; Table 1), suggesting that age is related to the development but not to the recurrence of prostate cancer. However, we observed statistically significant differences in PSA at diagnosis, pathologic Gleason grade group, clinical T stage, pathologic T stage, pathologic N stage, seminal vesicle invasion, perineural invasion, lymphovascular invasion, and D’Amico classification between the two groups of patients (*p* < 0.001; Table 1). A PSA of >10 ng/mL, pathologic Gleason grade groups 4 + 5, advanced tumor stages, lymph node metastasis, invasion, and a high-risk D’Amico classification appeared to be major risk factors for biochemical recurrence in these patients.

We further analyzed the genotype distributions of *MMP-11* polymorphisms in patients with prostate cancer. A previous study suggested that prostate cancers with high expression levels of *MMP-11* were significantly associated with a higher probability of biochemical recurrence [29]. Furthermore, a recent study by Escaff et al., indicated that MMPs, including *MMP-11*, were involved and played a crucial role in the tumorigenesis and biochemical recurrence of prostate cancer [30]. However, we observed no significant differences in the associations between biochemical recurrence and *MMP-11* polymorphisms among the five *MMP-11* SNPs selected in the present study (Table 2), suggesting that the direct impact of these SNPs on biochemical recurrence might be limited. Furthermore, we noted no significant associations between *MMP-11* polymorphisms and clinicopathological characteristics in the 578 patients with prostate cancer in this study. Notably, of the 175 patients with biochemical recurrence, those who carried the *MMP-11* rs131451 “TC + CC” polymorphic variants were associated with advanced clinical T stage (*p* = 0.007; OR: 3.238; 95% CI: 1.340–7.824; Table 8) and a high-risk D’Amico classification (*p* = 0.015, OR: 2.254, 95% CI: 1.164–4.364; Table 8) compared with those with the “TT” genotypes. Although the impact of *MMP-11* rs131451 on biochemical recurrence was low, previous research suggested that MMP-11 overexpression was associated with poor survival in patients with prostate cancer [18]. Thus, the *MMP-11* rs131451 “TC + CC” polymorphic variant may play a role in the development or regulation of biochemical recurrence in prostate cancer.

Previous studies have reported that *MMP-11* polymorphisms were associated with cancer risk and tumor development; however, the associations of the *MMP-11* SNPs with cancer susceptibility varied in different cancers [20,21,22,23,24]. No significant associations were observed among the *MMP-11* rs131451 polymorphic variants in patients with hepatocellular carcinoma [22] or uterine cervical cancer [23]. Conversely, patients with urothelial cell carcinoma who carried the *MMP-11* rs131451 polymorphic “CC” genotype were associated with a lower risk of later tumor T status (T1-T4) when compared with those who carried the CT + TT genotype [24]. Among the 175 patients with biochemical recurrence in the current study, those with the *MMP-11* rs131451 polymorphic “C” allele had a higher risk of later clinical T stage and high-risk D’Amico classification. This finding indicates the controversial role of *MMP-11* rs131451 polymorphisms in cancer development and biochemical recurrence in different cancers. A study conducted in Thailand revealed that *MMP-11* overexpression was significantly associated with poor survival and that it could potentially be used to predict poor prognosis in prostate cancer [18]. Furthermore, we used data from The Cancer Genome Atlas (TCGA) to analyze the relationship between *MMP-11* mRNA expression levels and prostate cancer carcinogenesis, clinicopathological characteristics, and biochemical recurrence [31]. The TCGA data analysis results revealed the *MMP-11* mRNA level was statistically significant different in clinical T stage (*p* = 0.0051), pathological T stage (*p* < 0.0001), pathological N stage (*p* < 0.0001), and biochemical recurrence (*p* < 0.0001). Taken together, these findings indicate that *MMP-11* rs131451 polymorphisms might be involved in the effect of *MMP-11* overexpression on both biochemical recurrence and poor prognosis in patients with prostate cancer.

One of the limitations of this study is the lack of tumor specimens from or information about *MMP-11* expression levels in patients with prostate cancer. A more detailed analysis comparing the effects of the different *MMP-11* genotypes and their mRNA and protein expression levels on prostate cancer tumor progression, biochemical recurrence, and disease prognosis is required.

## 5. Conclusions

In conclusion, our results demonstrate that the *MMP-11* polymorphisms, particularly rs131451, were associated with tumor development in prostate cancer patients with biochemical recurrence. Although the direct impact of *MMP-11* gene polymorphisms on the biochemical recurrence of prostate cancer was limited, patients with at least one polymorphic C allele (TC/CC) in rs131451 were associated with a higher risk of advanced-stage tumors and high-risk D’Amico classification compared with those with the wild-type homozygous (TT). The *MMP-11* SNP rs131451 may contribute to tumor development in prostate cancer patients with biochemical recurrence.

## Figures and Tables

**Table 1:** The distribution of demographic characteristics in 578 patients with prostate cancer.

Variable	Biochemical Recurrence		
	No (*n* = 403)	Yes (*n* = 175)	*p*-Value
**Age at diagnosis (years)**			
≤65	168 (41.7%)	77 (44.0%)	*p* = 0.605
>65	235 (58.3%)	98 (56.0%)	
**PSA at diagnosis (ng/mL)**			
≤10	218 (54.1%)	52 (29.7%)	*p* < 0.001 *
>10	185 (45.9%)	123 (70.3 %)	
**Pathologic Gleason grade group**			
1 + 2 + 3	366 (90.8%)	117 (66.9%)	*p* < 0.001 *
4 + 5	37 (9.2%)	58 (33.1%)	
**Clinical T stage**			
1 + 2	368 (91.3%)	132 (75.4%)	*p* < 0.001 *
3 + 4	35 (8.7%)	43 (24.6%)	
**Pathologic T stage**			
2	266 (66.0%)	40 (22.9%)	*p* < 0.001 *
3 + 4	137 (34.0%)	135 (77.1%)	
**Pathologic N stage**			
N0	392 (97.3%)	137 (78.3%)	*p* < 0.001 *
N1	11 (2.7%)	38 (21.7%)	
**Seminal vesicle invasion**			
No	363 (90.1%)	88 (50.3%)	*p* < 0.001 *
Yes	40 (9.9 %)	87 (49.7%)	
**Perineural invasion**			
No	140 (34.7%)	15 (8.6%)	*p* < 0.001 *
Yes	263 (65.3%)	160 (91.4%)	
**Lymphovascular invasion**			
No	372 (92.3%)	109 (62.3%)	*p* < 0.001 *
Yes	31 (7.7%)	66 (37.7%)	
**D’Amico classification**			
Low risk	55 (13.6%)	5 (2.9%)	*p* < 0.001 *
Intermediate risk	167 (41.4%)	52 (29.7%)	
High risk	181 (44.9%)	118 (67.4%)	

* *p*-value < 0.05 as statistically significant.

**Table 2:** Distribution frequency of *MMP-11* genotypes in 578 patients with prostate cancer.

Variable	Biochemical Recurrence		OR (95% CI)	AOR (95% CI)
	No (*n* = 403)	Yes (*n* = 175)		
**rs131451**				
TT	117 (29.0%)	58 (33.1%)	1.00	1.00
TC	209 (51.9%)	91 (52.0%)	0.878 (0.589–1.310)	0.727 (0.452–1.170)
CC	77 (19.1%)	26 (14.9%)	0.681 (0.395–1.174)	0.669 (0.348–1.286)
TC+CC	286 (71.0%)	117 (66.9%)	0.825 (0.564–1.208)	0.713 (0.453–1.121)
**rs738791**				
CC	189 (46.9%)	83 (47.4%)	1.00	1.00
CT	173 (42.9%)	81 (46.3%)	1.066 (0.737–1.542)	0.905 (0.580–1.412)
TT	41 (10.2%)	11 (6.3%)	0.611 (0.299–1.247)	0.615 (0.270–1.403)
CT+TT	214 (53.1%)	92 (52.6%)	0.979 (0.686–1.397)	0.849 (0.555–1.300)
**rs2267029**				
GG	195 (48.4%)	97 (55.4%)	1.00	1.00
GA	179 (44.4%)	69 (39.4%)	0.775 (0.536–1.121)	0.805 (0.518–1.251)
AA	29 (7.2%)	9 (5.2%)	0.624 (0.284–1.370)	0.638 (0.247–1.648)
GA+AA	208 (51.6%)	78 (44.6%)	0.754 (0.528–1.077)	0.782 (0.512–1.196)
**rs738792**				
TT	178 (44.2%)	85 (48.6%)	1.00	1.00
TC	186 (46.2%)	80 (45.7%)	0.901 (0.623–1.301)	0.961 (0.619–1.493)
CC	39 (9.7%)	10 (5.7%)	0.537 (0.256–1.127)	0.566 (0.232–1.381)
TC+CC	225 (55.8%)	90 (51.4%)	0.838 (0.587–1.195)	0.893 (0.584–1.366)
**rs28382575**				
TT	377 (93.5%)	164 (93.7%)	1.00	1.00
TC	26 (6.5%)	11 (6.3%)	0.973 (0.469–2.015)	1.240 (0.513–2.995)
CC	0 (0%)	0 (0.0%)	—	—
TC+CC	26 (6.5%)	11 (6.3%)	0.973 (0.469–2.015)	1.240 (0.513–2.995)

The odds ratios (ORs) and their 95% confidence intervals (CIs) were estimated by logistic regression models. The adjusted odds ratios (AORs) with their 95% confidence intervals (CIs) were estimated by multiple logistic regression models after controlling for age at diagnosis, PSA levels at diagnosis, pathologic Gleason grade group, clinical T stage, pathologic T stage, pathologic N stage, seminal vesicle invasion, perineural invasion, lymphovascular invasion, D’Amico classification, and biochemical recurrence.

**Table 3:** Odds ratio (OR) and 95% confidence interval (CI) of the clinicopathological characteristics and *MMP-11* rs131451 genotypic frequencies in 578 patients with prostate cancer.

Variable	Genotypic Frequencies			
rs131451	TT (*n* = 175)	TC + CC (*n* = 403)	OR (95% CI)	*p*-Value
**Pathologic Gleason grade group**				
1 + 2 + 3	150 (85.7%)	333 (82.6%)	1.00	*p* = 0.358
4 + 5	25 (14.3%)	70 (17.4%)	1.261 (0.768–2.070)	
**Clinical T stage**				
1 + 2	158 (90.3%)	342 (84.9%)	1.00	*p* = 0.080
3 + 4	17 (9.7%)	61 (15.1%)	1.658 (0.938–2.930)	
**Pathologic T stage**				
2	90 (51.4%)	216 (53.6%)	1.00	*p* = 0.631
3 + 4	85 (48.6%)	187 (46.4%)	0.917 (0.643–1.308)	
**Pathologic N stage**				
N0	163 (93.1%)	366 (90.8%)	1.00	*p* = 0.357
N1	12 (6.9%)	37 (9.2%)	1.373 (0.698–2.702)	
**Seminal vesicle invasion**				
No	135 (77.1%)	316 (78.4%)	1.00	*p* = 0.735
Yes	40 (22.9%)	87 (21.6%)	0.929 (0.607–1.422)	
**Perineural invasion**				
No	47 (26.9%)	108 (26.8%)	1.00	*p* = 0.988
Yes	128 (73.1%)	295 (73.2%)	1.003 (0.672–1.497)	
**Lymphovascular invasion**				
No	149 (85.1%)	332 (82.4%)	1.00	*p* = 0.414
Yes	26 (14.9%)	71 (17.6%)	1.226 (0.751–1.999)	
**D’Amico classification**				
Low/Intermediate risk	86 (49.1%)	193 (47.9%)	1.00	*p* = 0.782
High risk	89 (50.9%)	210 (52.1%)	1.051 (0.737–1.499)	

The ORs analyzed by their 95% CIs were estimated by logistic regression models.

**Table 4:** Odds ratio (OR) and 95% confidence interval (CI) of the clinicopathological characteristics and *MMP-11* rs738791 genotypic frequencies in 578 patients with prostate cancer.

Variable	Genotypic Frequencies			
rs738791	CC (*n* = 272)	CT + TT (*n* = 306)	OR (95% CI)	*p*-Value
**Pathologic Gleason grade group**				
1 + 2 + 3	233 (85.7%)	250 (81.7%)	1.00	*p* = 0.199
4 + 5	39 (14.3%)	56 (18.3%)	1.338 (0.857–2.091)	
**Clinical T stage**				
1 + 2	233 (85.7%)	267 (87.3%)	1.00	*p* = 0.576
3 + 4	39 (14.3%)	39 (12.7%)	0.873 (0.541–1.407)	
**Pathologic T stage**				
2	153 (56.3%)	153 (50.0%)	1.00	*p* = 0.133
3 + 4	119 (43.8%)	153 (50.0%)	1.286 (0.926–1.785)	
**Pathologic N stage**				
N0	250 (91.9%)	279 (91.2%)	1.00	*p* = 0.751
N1	22 (8.1%)	27 (8.8%)	1.100 (0.611–1.980)	
**Seminal vesicle invasion**				
No	216 (79.4%)	235 (76.8%)	1.00	*p* = 0.449
Yes	56 (20.6%)	71 (23.2%)	1.165 (0.784–1.732)	
**Perineural invasion**				
No	75 (27.6%)	80 (26.1%)	1.00	*p* = 0.699
Yes	197 (72.4%)	226 (73.9%)	1.076 (0.744–1.555)	
**Lymphovascular invasion**				
No	231 (84.9%)	250 (81.7%)	1.00	*p* = 0.300
Yes	41 (15.1%)	56 (18.3%)	1.262 (0.812–1.961)	
**D’Amico classification**				
Low/Intermediate risk	136 (50.0%)	143 (46.7%)	1.00	*p* = 0.433
High risk	136 (50.0%)	163 (53.3%)	1.140 (0.822–1.581)	

The ORs analyzed by their 95% CIs were estimated by logistic regression models.

**Table 5:** Odds ratio (OR) and 95% confidence interval (CI) of the clinicopathological characteristics and *MMP-11* rs2267029 genotypic frequencies in 578 patients with prostate cancer.

Variable	Genotypic Frequencies			
rs2267029	GG (*n* = 292)	GA + AA (*n* = 286)	OR (95% CI)	*p*-Value
**Pathologic Gleason grade group**				
1 + 2 + 3	242 (82.9%)	241 (84.3%)	1.00	*p* = 0.652
4 + 5	50 (17.1%)	45 (15.7%)	0.904 (0.582–1.404)	
**Clinical T stage**				
1 + 2	248 (84.9%)	252 (88.1%)	1.00	*p* = 0.263
3 + 4	44 (15.1%)	34 (11.9%)	0.760 (0.470–1.230)	
**Pathologic T stage**				
2	148 (50.7%)	158 (55.2%)	1.00	*p* = 0.272
3 + 4	144 (49.3%)	128 (44.8%)	0.833 (0.600–1.155)	
**Pathologic N stage**				
N0	268 (91.8%)	261 (91.3%)	1.00	*p* = 0.822
N1	24 (8.2%)	25 (8.7%)	1.070 (0.596–1.921)	
**Seminal vesicle invasion**				
No	222 (76.0%)	229 (80.1%)	1.00	*p* = 0.241
Yes	70 (24.0%)	57 (19.9%)	0.789 (0.532–1.172)	
**Perineural invasion**				
No	74 (25.3%)	81 (28.3%)	1.00	*p* = 0.419
Yes	218 (74.7%)	205 (71.7%)	0.859 (0.594–1.242)	
**Lymphovascular invasion**				
No	245 (83.9%)	236 (82.5%)	1.00	*p* = 0.656
Yes	47 (16.1%)	50 (17.5%)	1.104 (0.714–1.709)	
**D’Amico classification**				
Low/Intermediate risk	135 (46.2%)	144 (50.3%)	1.00	*p* = 0.322
High risk	157 (53.8%)	142 (49.7%)	0.848 (0.612–1.175)	

The ORs analyzed by their 95% CIs were estimated by logistic regression models.

**Table 6:** Odds ratio (OR) and 95% confidence interval (CI) of the clinicopathological characteristics and *MMP-11* rs738792 genotypic frequencies in 578 patients with prostate cancer.

Variable	Genotypic Frequencies			
rs738792	TT (*n* = 263)	TC + CC (*n* = 315)	OR (95% CI)	*p*-Value
**Pathologic Gleason grade group**				
1 + 2 + 3	220 (83.7%)	263 (83.5%)	1.00	*p* = 0.959
4 + 5	43 (16.3%)	52 (16.5%)	1.012 (0.650–1.574)	
**Clinical T stage**				
1 + 2	224 (85.2%)	276 (87.6%)	1.00	*p* = 0.391
3 + 4	39 (14.8%)	39 (12.4%)	0.812 (0.503–1.308)	
**Pathologic T stage**				
2	129 (49.0%)	177 (56.2%)	1.00	*p* = 0.087
3 + 4	134 (51.0%)	138 (43.8%)	0.751 (0.540–1.043)	
**Pathologic N stage**				
N0	242 (92.0%)	287 (91.1%)	1.00	*p* = 0.698
N1	21 (8.0%)	28 (8.9%)	1.124 (0.623–2.030)	
**Seminal vesicle invasion**				
No	220 (76.0%)	251 (79.7%)	1.00	*p* = 0.293
Yes	63 (24.0%)	64 (20.3%)	0.809 (0.546–1.201)	
**Perineural invasion**				
No	65 (24.7%)	90 (28.6%)	1.00	*p* = 0.297
Yes	198 (75.3%)	225 (71.4%)	0.821 (0.566–1.190)	
**Lymphovascular invasion**				
No	222 (84.4%)	259 (82.2%)	1.00	*p* = 0.483
Yes	41 (15.6%)	56 (17.8%)	1.171 (0.753–1.820)	
**D’Amico classification**				
Low/Intermediate risk	122 (46.4%)	157 (49.8%)	1.00	*p* = 0.408
High risk	141 (53.6%)	158 (50.2%)	0.871 (0.627–1.209)	

The ORs analyzed by their 95% CIs were estimated by logistic regression models.

**Table 7:** Odds ratio (OR) and 95% confidence interval (CI) of the clinicopathological characteristics and *MMP-11* rs28382575 genotypic frequencies in 578 patients with prostate cancer.

Variable	Genotypic Frequencies			
rs28382575	TT (*n* = 541)	TC + CC (*n* = 37)	OR (95% CI)	*p*-Value
**Pathologic Gleason grade group**				
1 + 2 + 3	453 (83.7%)	30 (81.1%)	1.00	*p* = 0.674
4 + 5	88 (16.3%)	7 (18.9%)	1.201 (0.511–2.821)	
**Clinical T stage**				
1 + 2	468 (86.5%)	32 (86.5%)	1.00	*p* = 0.997
3 + 4	73 (13.5%)	5 (13.5%)	1.002 (0.378–2.654)	
**Pathologic T stage**				
2	281 (51.9%)	25 (67.6%)	1.00	*p* = 0.065
3 + 4	260 (48.1%)	12 (32.4%)	0.519 (0.255–1.054)	
**Pathologic N stage**				
N0	495 (91.5%)	34 (91.9%)	1.00	*p* = 0.934
N1	46 (8.5%)	3 (8.1%)	0.949 (0.281–3.211)	
**Seminal vesicle invasion**				
No	419 (77.4%)	32 (86.5%)	1.00	*p* = 0.199
Yes	122 (22.6%)	5 (13.5%)	0.537 (0.205–1.407)	
**Perineural invasion**				
No	143 (26.4%)	12 (32.4%)	1.00	*p* = 0.425
Yes	398 (73.6%)	25 (67.6%)	0.749 (0.366–1.529)	
**Lymphovascular invasion**				
No	450 (83.2%)	31 (83.8%)	1.00	*p* = 0.924
Yes	91 (16.8%)	6 (16.2%)	0.957 (0.388–2.361)	
**D’Amico classification**				
Low/Intermediate risk	262 (48.4%)	17 (45.9%)	1.00	*p* = 0.770
High risk	279 (51.6%)	20 (54.1%)	1.105 (0.566–2.155)	

The ORs analyzed by their 95% CIs were estimated by logistic regression models.

**Table 8:** Odds ratio (OR) and 95% confidence interval (CI) of the clinicopathological characteristics and *MMP-11* rs131451 genotypic frequencies in 175 patients with prostate cancer with biochemical recurrence.

Variable	Genotypic Frequencies			
rs131451	TT (*n* = 58)	TC + CC (*n* = 117)	OR (95% CI)	*p*-Value
**Pathologic Gleason grade group**				
1 + 2 + 3	42 (72.4%)	75 (64.1%)	1.00	*p* = 0.272
4 + 5	16 (27.6%)	42 (35.9%)	1.470 (0.738–2.927)	
**Clinical T stage**				
1 + 2	51 (87.9%)	81 (69.2%)	1.00	*p* = 0.007 *
3 + 4	7 (12.1%)	36 (30.8%)	3.238 (1.340–7.824)	
**Pathologic T stage**				
2	13 (22.4%)	27 (23.1%)	1.00	*p* = 0.922
3 + 4	45 (77.6%)	90 (76.9%)	0.963 (0.454–2.043)	
**Pathologic N stage**				
N0	48 (82.8%)	89 (76.1%)	1.00	*p* = 0.312
N1	10 (17.2%)	28 (23.9%)	1.510 (0.677–3.370)	
**Seminal vesicle invasion**				
No	32 (55.2%)	56 (47.9%)	1.00	*p* = 0.363
Yes	26 (44.8%)	61 (52.1%)	1.341 (0.713–2.522)	
**Perineural invasion**				
No	5 (8.6%)	10 (8.5%)	1.00	*p* = 0.987
Yes	53 (91.4%)	107 (91.5%)	1.009 (0.328–3.103)	
**Lymphovascular invasion**				
No	39 (67.2%)	70 (59.8%)	1.00	*p* = 0.341
Yes	19 (32.8%)	47 (40.2%)	1.378 (0.711–2.670)	
**D’Amico classification**				
Low/Intermediate risk	26 (44.8%)	31 (26.5%)	1.00	*p* = 0.015 *
High risk	32 (55.2%)	86 (73.5%)	2.254 (1.164–4.364)	

The ORs analyzed by their 95% CIs were estimated by logistic regression models. * *p*-value < 0.05 as statistically significant.
